# Mean Platelet Volume as a Negative Marker of Inflammation in Children with Rotavirus Gastroenteritis

**Published:** 2014-10-28

**Authors:** Celik Tanju, Güler Ekrem, Atas Berksoy Emel, Arslan Nur

**Affiliations:** 1 Department of Pediatric Emergency, Dr. Behcet Uz Children’s Hospital, Izmir; 2 Department of Pediatric Emergency, Dr. Sütçü Imam University, Faculty of Medicine, Maras; 3 Division of Pediatric Gastroenterology, Dokuz Eylul University, Faculty of Medicine, Hepatology and Nutrition, Izmir, Turkey

**Keywords:** Child, Gastroenteritis, Inflammation, Mean Platelet Volume, Rotavirus

## Abstract

***Objective:*** Mean platelet volume (MPV) is a determinant of inflammation. The aim of the present study was to investigate the MPV levels in children with rotavirus gastroenteritis and to evaluate the possible relationship between MPV and severity of gastroenteritis.

***Methods:*** Children diagnosed with acute rotavirus gastroenteritis and healthy controls were enrolled in this study. Patients were classified into three disease severity groups based on their Vesikari score (<7 mild, 7-10 moderate and >11 severe). Rotavirus was determined in fresh stool samples using ELISA test. Leukocyte and platelet counts, MPV and C-reactive protein (CRP) levels were assessed for all children.

***Findings:*** A total of 151 patients with rotavirus gastroenteritis (mean age 2.41± 0.14 years) and 80 healthy controls (mean age 2.63±0.22 years, *P*=0.129) were enrolled. MPV levels of children with rotavirus gastroenteritis were significantly lower than those of healthy peers (7.48±0.04 vs 7.79±0.07 fl, *P*=0.000). MPV levels were not significantly different among three gastroenteritis groups. Gastroenteritis score was positively correlated with leukocyte (*r*=0.670, *P*<0.01) and platelet count (*r*=0.159, *P*<0.05) and CRP level (*r*=0.256, *P*<0.01) in patients group. MPV was inversely correlated with platelet count. There was no significant correlation between MPV and gastroenteritis score.

***Conclusion:*** MPV levels were significantly lower in children with rotavirus gastroenteritis compared to controls. MPV can be used as a negative acute phase reactant in children with rotavirus gastroenteritis.

## Introduction

Infectious gastroenteritis is the most common cause of morbidity and mortality worldwide, especially in children younger than 5 years old^[^^1^^]^. Rotavirus is a leading cause of infectious diarrhea in children worldwide, followed by adenovirus and norovirus^[^^1^^]^. Rotavirus infections are known to be more severe and more often associated with a complicated course in viral etiologies^[^^2^^]^. Severe rotavirus-related mortality has been reported in younger children in particular^[^^3^^]^. Moreover, rotavirus may also be the cause of viremia^[^^4^^]^ and several other systemic disorders including pancreatitis^[^^5^^]^, cerebellitis^[^^6^^,^^7^^]^, systemic inflammatory response syndrome^[^^8^^]^, toxic megacolon^[^^9^^]^ and disseminated intra-vascular coagulation^[^^8^^,^^10^^]^. 

 Platelets are intimately involved in homeostasis, inflammation, immunity, tissue regeneration and other physiological and pathological processes^[^^11^^]^. Platelets play an important role in the pathogenesis of disorders associated with local or systemic inflammation^[^^11^^,^^12^^]^. Thrombotic and inflamma-tory agents, released from platelets, may trigger disease-specific complications^[^^13^^,^^14^^]^. The mean platelet volume (MPV) is a marker of platelet function and activation. Large platelets are hemostatically more active^[^^15^^]^. MPV has long been recognized as an inflammatory marker and its role has been previously demonstrated in various gastrointestinal and other systemic disorders such as familial Mediterranean fever^[^^16^^]^, fatty liver disease and athero-sclerosis^[^^17^^,^^18^^]^, renal diseases^[^^19^^]^, inflammatory bowel disease^[^^20^^]^, liver disease^[^^21^^] ^and amebiasis^[^^22^^]^. 

 To our knowledge, there is only one study assessing MPV levels in patients with acute rotavirus gastroenteritis^[^^23^^]^. Therefore, the aim of the present study was to investigate the MPV levels in children with acute gastroenteritis caused by rotavirus and to compare the MPV levels with those of healthy controls. The second aim of this study was to evaluate the possible relationship between MPV and severity of gastroenteritis in patients.

## Subjects and Methods

This is a case-control study conducted in patients diagnosed with rotavirus gastroenteritis in Dr. Behcet Uz Children Hospital, Pediatric Emergency Department between January and May 2013. 151 patients with rotavirus gastroenteritis (65 females, mean age 2.41± 0.14 years, 6 months to 6 years) were enrolled in the study. Patients who had chronic disease, malabsorption syndromes, immunodeficiency and malnutrition, as well as those who used medication were not included. Hospitalized patients were also excluded. The control group consisted of 80 sex- and age-matched children (36 females, mean age 2.63±0.22 years, 6 months to 6 years) who were brought to the well child outpatient clinic for medical control (screening for hepatitis, thyroid functions or anemia, etc.). None had a history of chronic disease or medication usage. Physical examination of all patients and controls was performed.

 The complete blood count analyses were performed in the same Coulter analyzer (LH-780, Beckman Coulter, Brea, CA, USA) with the impedance method (intra-assay variation coefficient 1.6%, interassay variation coefficient 1.65%), which was routinely checked every month in the central laboratory of our institution in regular intervals of one hour. Standard tubes with constant amount of K3-EDTA were used for complete blood count analyses. 

 Rotavirus was determined in fresh stool samples using the highly sensitive and specific ELISA test (Rota Adeno Antigen Test Device, Cambridge). In addition, stool cultures and antigen tests were assessed for potential causative bacterial agents (Salmonella, Shigella, Campylobacter) and parasitic etiologies (*Entamoeba histolytica*). 

 The severity of diarrhea in patients was assessed according to Vesikari scoring system based on the duration and peak frequency of diarrhea and vomiting, the extent of fever and the severity of dehydration^[^^24^^]^. Patients were classified into three disease severity groups based on their Vesikari score (<7 mild, 7-10 moderate and >11 severe)^[^^24^^]^.

 C-reactive protein, leukocyte and thrombocyte counts, mean platelet volume, blood urea nitrogen and creatinine levels of patients and controls were compared. 

 The study protocol was designed in compliance with the Declaration of Helsinki. Informed consent was obtained from parents on enrollment in the study. The study was started after the approval of the Ethics Committee of the Sütçü Imam University, Faculty of Medicine. 

 Statistical analysis was performed using the Statistical Package for Social Sciences (SPSS) version 15.0 (SPSS, Inc., Chicago, IL, USA). Data were expressed as mean±standard errors of mean. Kolmogorov-Smirnov test was used to examine normal distribution of the population. One way analysis of variance was used for analyzing group averages among three groups. Student *t* test and Mann-Whitney U-test were used for comparing two group averages as a post hoc test. Chi-square test was used for comparing group ratios.

**Table 1:** Comparison of clinical and laboratory parameters between patients and controls (mean±SEM)

	**Gastroenteritis group (n=151)**	**Control group** **(n=80)**	***P-*** **value**
**Age (years)**	2.41 (0.14)	2.63 (0.22)	0.1
**Gender (M/F)**	86 / 65	44 / 36	0.8
**Leukocyte (count/mm** ^3^ **)**	11000 (370)	8900 (290)	<0.001
**Hemoglobin (g/dl)**	11.70 (0.11)	11.48 (0.19)	0.1
**Platelet (count/mm** ^3^ **)**	371 500 (10 500)	316 000 (10 000)	<0.001
**MPV (fl)**	7.48 (0.04)	7.79 (0.07)	<0.001
**C reactive protein (mg/dl)**	0.95 (0.15)	0.29 (0.01)	<0.001
**Blood urea nitrogen (mg/dl)**	12.44 (0.46)	9.24 (0.47)	<0.001
**Creatinine (mg/dl)**	0.39 ( 0.01)	0.38 (0.02)	0.8

Inter-correlations between parameters were computed through the Pearson’s correlation analysis. Correlation coefficient indicated low correlation at 0.10–0.29, medium correlation at 0.30–0.49, and high correlation at 0.50. All *P-*values were two-tailed and group differences or correlations with *P*<0.05 were considered to be statistically significant.

## Findings

The clinical and laboratory characteristics of the patients and controls are summarized in Table I. There were no significant differences between the groups regarding age and gender (*P*=0.129 and *P*=0.728, respectively) (Table 1). Leukocyte and platelet counts, C-reactive protein and blood urea nitrogen levels of the patients were higher than those of controls (Table 1).

 MPV levels showed no difference between girls and boys (7.63±0.06 vs 7.55±0.05 fl, *P*=0.292). MPV levels of patients with rotavirus gastroenteritis were significantly lower than those of control children (7.48±0.04 vs 7.79±0.07 fl, *P*=0.000) (Table 1). When the patients were classified into clinically mild, moderate and severe gastroenteritis groups according to Vesikari system, MPV did not show a significant difference among these subgroups (*P*>0.05) (Table 2). 

Gastroenteritis score was positively correlated with leukocyte and platelet count and CRP level in patient’s group (Table 3). MPV was negatively correlated with thrombocyte count. There was no significant correlation between MPV and gastroenteritis score (Table 3).

**Table 2:** Comparison of laboratory characteristics of the patients classified according to Vesikari clinical severity scoring system (mean±SEM)

**Severity of gastroenteritis**				
	**Mild (n=21)**	**Moderate (n=53)**	**Severe (n=77)**	***P-*** **value**
**Leukocyte (count/mm** ^3^ **)**	8000 (470)	8200 (370)	13800 (500)	Mi-Mo (0.753) Mi-S (0.000) Mo-S (0.000)
**Platelet (count/mm** ^3^ **)**	369000 (22400)	341000 (20100)	392000 (13300)	Mi-Mo (0.447) Mi-S (0.408) Mo-S (0.043)
**MPV (fl)**	7.42 (0.11)	7.53 (0.08)	7.46 (0.05)	Mi-Mo (0.464) Mi-S (0.695) Mo-S (0.484)
**C reactive protein (mg/dl)**	0.66 (0.31)	0.68 (0.12)	1.21 (0.32)	Mi-Mo (0.955) Mi-S (0.328) Mo-S (0.101)
**Blood urea nitrogen (mg/dl)**	7.41 (1.01)	11.84 (0.68)	13.97 (0.62)	Mi-Mo (0.001) Mi-S (0.000) Mo-S (0.027)
**Creatinine (mg/dl)**	0.28 (0.02)	0.41 (0.02)	0.40 (0.01)	Mi-Mo (0.002) Mi-S (0.003) Mo-S (0.811)

**Table 3:** Correlation between laboratory parameters in patients

**Parameter**	**Leuko-cyte**	**MPV**	**Platelet**	**Gastroenteritis score**
**Mean Platelet Volume**	0.059			
**Platelet**	0.388**	-0.204**		
**Gastroenteritis score**	0.670**	-0.015	0.159*	
**C-Reactive Protein**	0.248**	-0.078	-0.052	0.256**

* Correlation is significant at the 0.05 level

** Correlation is significant at the 0.01 level

## Discussion

This study demonstrated that MPV levels of children with rotavirus gastroenteritis were significantly lower than the thoe of healthy controls. On the other hand, the difference between MPV levels among three clinical patient groups did not reach a statistical significance. There are only two studies investigating MPV levels in patients with acute gastroenteritis in the literature^[^^22^^,^^23^^]^. Matowicka-Karna et al^[^^22^^]^ found that MPV levels in patients infested with *Entamoeba histolytica *were lower than in controls. Similarly, Mete et al^[^^23^^]^ recently showed that MPV levels were lower in children with rotavirus gastroenteritis than the in healthy peers. Moreover, they did not find any difference between two disease severity groups regarding MPV levels^[^^23^^]^. All of these findings described by Mete et al are similar to our results. MPV acts as a negative or positive acute phase reactant in different inflammatory conditions depending on the severity of the systemic inflammation^[^^25^^]^. It has been hypothesized that MPV might increase in low-grade inflammation owing to the presence of the large platelets in the circulation and might decrease in more severe inflammation due to the consumption of these large platelets in the vascular segments of the inflammatory region ^[^^23^^,^^25^^,^^26^^]^. Decreased MPV levels had been especially showed in inflammatory gastrointestinal diseases, such as inflammatory bowel disease^[^^20^^,^^27^^,^^28^^]^, acute appendicitis^[^^29^^,^^30^^]^, acute gastroenteritis^[^^22^^,^^23^^]^ and intestinal tuber-culosis^[^^31^^]^. Although the pathogenesis of this decrease in MPV in intestinal inflammation has not been fully explained, it seems reasonable to explain this with the sequestration of large active platelets in the vascular segments of the inflamed bowel. New studies including larger patient groups and investigating the possible mechanisms are needed for this topic. 

 In this study MPV levels were negatively correlated with thrombocyte count. Although both the platelet count and MPV level are accepted as acute phase reactants, this inverse relationship is frequently described in some pathological conditions and it reflects the effort to maintain homeostasis by preserving a constant platelet mass^[^^16^^,^^17^^,^^23^^,^^25^^,^^32^^]^. This inverse relation-ship can be caused by consumption of large platelets in severe inflammatory conditions, defective thrombopoiesis or by swelling of circulating platelets in an environment rich in activating agents^[^^25^^]^. Future prospective studies are needed to clarify the clinical importance of these conflicting results.

 One major limitation of our study is the cross-sectional, case-control design of the study. A prospective study which analyzes the alterations of MPV before and after rehydration therapy is needed. Another limitation is the lack of the other parasitic and bacterial gastroenteritis group in this study. It is known that patients with bacterial or parasitic diarrhea have higher inflammatory signs (higher CRP levels, leukocytosis etc)^[^^2^^]^. Similarly, higher MPV levels of these patient groups can be expected.

## Conclusion

In this study, we found decreased levels of MPV in patients with rotavirus gastroenteritis when compared with healthy children. Since MPV is a simple laboratory parameter, we suggest its use as a negative acute phase reactant in rotavirus gastroenteritis.
